# An Experimental Approach for Investigating Fatigue-Induced Debonding Propagation in Composite Stiffened Panels Using Thermographic Phase Mapping

**DOI:** 10.3390/polym17020181

**Published:** 2025-01-14

**Authors:** Aniello Riccio, Angela Russo, Cinzia Toscano, Mauro Zarrelli

**Affiliations:** 1 Department of Engineering, University of Campania Luigi Vanvitelli, Via Roma 29, 81030 Aversa, CE, Italy; aniello.riccio@unicampania.it; 2 Institute for Polymers Composites and Biomaterials, Italian National Research Council, Piazzale Enrico Fermi, 80055 Portici, NA, Italy; mauro.zarrelli@cnr.it; 3 CIRA Italian Aerospace Research Centre, Via Maiorise s/n, 81043 Capua, CE, Italy; c.toscano@cira.it

**Keywords:** fatigue, experimental method, lock-in thermography, skin-stringer debonding, thermographic phase maps, stiffened panel

## Abstract

This work introduces an experimental approach focused on investigating fatigue-driven debonding in a composite structure designed to simulate the complexity of a typical aeronautical panel. The debonding is placed between the skin and the stringer, and the structure has been tested under fatigue compression conditions. Using lock-in thermography, the damage evolution during fatigue cycles has been detailed monitored. Indeed, thermographic phase maps obtained after a predetermined number of cycles during the whole fatigue test have been graphically analysed and have allowed us to obtain an accurate measurement of the delaminated area extent and shape. Our approach advances the understanding of damage propagation in composite materials, contributing to the development of damage-tolerant structural designs and supplying valuable data to validate numerical fatigue prediction models. Furthermore, the use of non-destructive testing techniques, such as thermography, has been found crucial for accurately quantifying the extent and the shape of the debonding after a given number of fatigue cycles.

## 1. Introduction

The study of fatigue damage propagation in composite materials structures is still an understudied topic. Experimental tests to understand the evolution of fatigue damage are complex and expensive. This is not only due to the manufacturing costs of the structures, but mainly due to the time needed, as a complex structure can take weeks or even months to deteriorate under fatigue cycles. In some cases, depending on the cyclic load applied, the structure may even have an infinite fatigue life. For this reason, experiments are generally performed on small coupons to characterise the behaviour of the material rather than the overall structure. Experimental tests to assess the fatigue behaviour of complex composite structures are not as common, but at the same time essential for a proper design. A direct consequence of this criticality is the limited availability of robust and efficient numerical methodologies for the simulation of composite fatigue failure. Indeed, in order to validate and verify numerical models, experimental comparison is required, which cannot be limited to coupons or the number of cycles to failure. Rather, verification of the fatigue life history of a component subjected to a given cyclic loading condition is necessary. This is the context for this paper, where a novel experimental approach using thermographic phase maps has been proposed to investigate the propagation of fatigue delamination in composite stiffened panels. The proposed approach allowed us to obtain accurate images of the damage propagation with the fatigue cycles in a typical composite aircraft structure. In particular, a T-shaped stiffener has been selected as a subcomponent structure based on its relevance to aerospace engineering, specifically in the context of damage tolerance design. Indeed, the T-shaped stiffener is a widely used structural element in aeronautical applications, particularly in skin–stringer configurations, where delamination at the interface represents a critical failure mode under cyclic loading [1,2].

The study focuses on this configuration to contribute to the understanding of delamination propagation mechanisms and provide experimental data for validating predictive models. A rich state-of-the-art model exists on fatigue in composite materials, particularly on experimental tests at the coupon level.

Fatigue stress in composite structures can cause several effects, including reduction in structural strength, localised damage such as delaminations and micro-cracking, and gradual accumulation of damage in the material. This damage can be influenced by different factors, such as fibre orientation, areas of high-stress concentration, and environmental conditions like temperature and humidity. To manage these effects, structures need to be properly designed, and the structural integrity should be continuously monitored over time. This may include the use of advanced analyses and fatigue tests on representative samples of the structure. Many authors have experimentally studied the fatigue degradation of composite materials under different types of loads and boundary conditions. In [3], the effects of thermal cycling on the structural health of polyester matrix composites have been studied using a large-scale composite model. Damage to the composites during the heating and cooling phases has been observed using equipment specially designed for thermal cycling. In [4], Double Cantilever Beam (DCB) samples manufactured from a graphite/epoxy prepreg (G0814/913) containing a through-notch have been tested under constant amplitude fatigue. In the article in [5], an extensive numerical and experimental investigation of the fracture mechanical properties of end-notched flexural (ENF) specimens has been conducted. Static and cyclic properties have been studied for different interfaces and graphite/epoxy composites. The study in [6,7] examines the tensile-tensile fatigue behaviour of quasi-isotropic glass/epoxy laminates. Constant-amplitude tensile–tensile fatigue tests have been performed at different stress levels with a stress ratio. Damage growth in the material has been characterised by evaluating stiffness degradation. In [8], the failure mechanisms of composites with fibre waviness and ply drops under compressive fatigue loading have been investigated.

The presence of pre-existing damage can significantly affect the residual strength of composite material structures. Cracks or delaminations can act as stress concentration points, accelerating the crack propagation process and reducing the in-service life of the structure. Moreover, damage can lead to increased sensitivity to fatigue, with the risk of premature structural failure. In a damage-tolerant design philosophy, the testing of damaged structures under fatigue loading conditions is needed to assess how the damage affects the residual structural strength and the life of the components. The study in [9] experimentally investigates the damage tolerance of typical omega-stringer stiffened composite panels, with an initial delamination between the skin and the stringer foot under fatigue in the post-buckling regime. In [10], fatigue tests have been carried out to estimate the evolution of the defect in different impacted composite specimens. The paper in [11] presents a study on composite stiffened boxes under axial compression and torque, applied individually and in combination, both statically and cyclically.

A potential issue that can occur in aerospace structures is the separation between skin and stringer. This phenomenon can be caused by various factors, such as high loads, impacts, defects in the manufacturing process, or fatigue stresses, leading to a reduction in the load-bearing capacity of the structure and increasing the risk of premature structural failure. Quality control during the manufacturing process and structural integrity assessment through non-destructive testing are needed to prevent skin–stringer debonding. In addition, constant monitoring of the condition of the structure during its operational life is essential. The debonding evolution between skin and stringer in composite stiffened panels has been extensively studied in the literature. In [12], the skin-omega stringer separation has been studied, considering different load cases and allowing debonding to start in diverse locations. In [13], tests have been performed on samples consisting of a conical composite flange, representing a beam or frame, bonded to a composite skin. The tests have been carried out under monotonic loading conditions in tension, three-point bending, and combined tension/bending to evaluate the separation mechanisms between the skin and the bonded stringer. The paper in [14] introduces a study on the debonding growth between skin and lattice stiffeners using both numerical and experimental procedures. In [15], the fatigue life and the damage tolerance of composite stiffened panels with pre-existing debonding are investigated experimentally using single omega-stringer compression specimens. The influence of hygrothermal aging on co-bonded composite stiffened panels with an initial disbond under cyclic compression loading is investigated in [16]. However, there is a notable scarcity of detailed experimental measurements of the extent and shape of the fatigue-driven delamination propagation in complex composite structures.

Non-destructive Testing (NDT) is a key part of damage analysis of composite aircraft structures. Among the most commonly used NDT methods are X-ray tomography, which can detect internal defects such as resin inclusions, air bubbles, or fibre fractures, and ultrasound, which uses high-frequency sound waves to detect changes in material density. The latter method is particularly effective in identifying delaminations. Another widely used approach is active infrared thermography, which detects subsurface defects by differences in the surface temperature of a material in which a thermal gradient with respect to the environment is induced. The paper in [17] discusses the detection of damage in hybrid natural fibre composite laminates using the active thermography method. Specimens have been subjected to impact loading and post-impact fatigue loading to study damage progression through thermography. In [18], test samples have been produced and tested by impact tests to simulate the effect of stone fragments. Subsequently, the impacted samples were analysed by thermography.

Thermography can also be used during fatigue tests. Indeed, during the loading cycle, areas showing thermal anomalies may indicate the presence of evolving structural damage. For example, delaminations are prone to heat accumulation during the loading cycle due to relative movement between composite layers, while microcracks may generate localised heat due to stress concentration. In the paper in [19], a thermographic approach, originally developed for metal alloys, has been employed to rapidly determine the high-cycle fatigue strength of the composite. In [20], lock-in thermography has been used to study the repair efficiency of a bonded aerospace composite subjected to a step-wise cyclic mechanical load. Other relevant studies on the use of thermography to monitor damage evolution are reported in [21,22,23,24].

The main difficulty encountered in the literature is the identification of studies analysing representative structures of composite sub-components that provide a clear and quantitative description of the damage evolution under cyclic loading. This study aims to address this gap by contributing valuable insights. The results presented not only enhance the understanding of the fatigue behaviour of such structures but also serve to validate models and methodologies.

### Objective of the Research

Composite materials, due to their anisotropic nature, experience complex failure modes when subjected to compressive stresses, including matrix cracking, delamination, and fibre breakage. These phenomena are especially critical in aerospace applications, where compressive loads are common. By studying compressive fatigue, a deeper understanding can be gained of how these failure modes evolve under repeated compressive loads, providing insight into the behaviour of materials under real-world conditions. In addition, this research is critical to improving material failure prediction and fatigue life estimation of composite components. Understanding how damage accumulates under compressive fatigue allows more accurate predictive models to be developed, helping to estimate the service life of composite structures more reliably.

Thermographic phase maps are an advanced representation of data obtained through modulated thermography techniques, such as lock-in thermography. These maps are used to identify and analyse defects or anomalies within materials, offering specific advantages over simple thermal imaging. In this study, an experimental approach using the thermographic phase maps obtained through a lock-in thermography system has been used to monitor the evolution of the fatigue-driven debonding between skin and stringer within a pre-damaged composite structure during a load-controlled test. A panel containing a single stringer has been manufactured using a carbon fibre-reinforced polymer material system. The panel and the stringer have been subjected to an autoclave co-cure process. However, a large release area has been deliberately introduced using a Teflon sheet. Then, the panel was subjected to a compression–compression fatigue test to assess the evolution of the separation between skin and stringer. The lock-in thermographic system was been positioned in front of the specimen, mounted in the testing machine, and the tests were stopped at different fatigue cycles to capture two images of the specimen under maximum and minimum load, respectively. After the test, a graphical analysis technique based on thermographic images has been adopted to calculate the extension of the delaminated area.

The main contribution of this work to the state of the art lies in the advanced processing of thermographic images, aimed at visualising in detail the evolution of delamination during different fatigue cycles. This methodology allows not only to monitor the progress of the phenomenon but also to precisely quantify the delaminated area. Such data are fundamental to understanding the propagation mechanisms of fatigue-induced delamination and represent an essential basis for the validation of numerical methodologies, such as the finite element simulation of the phenomenon, as described in [25].

Section 2 contains the description of the investigated panel, while Section 3 includes its manufacturing. In Section 3, the proposed experimental approach is described, including the pre-crack test and the fatigue test description. Finally, in Section 4, the details on the calculation of the debonded area are provided. Conclusive remarks are given in Section 5.

## 2. Specimen Description

The panel investigated in this research is a demonstrative structure of a subcomponent of a wing box. More precisely, it is a typical aeronautical stiffened panel characterised by a single stringer with a T-shaped cross-section. Intralaminar damage has been deliberately introduced in the panel, consisting of a debonding between the skin and the reinforcement, whose dimensions have been selected through a preliminary numerical analyses campaign, in order to obtain a structure that highlights specific fatigue delamination growth criticalities, such as unstable propagation. Indeed, the analysis of artificially damaged structures is a common practice in structural engineering and scientific research. The main purpose has been to evaluate the propagation of damage under service loads, which, although considerably less than the critical buckling and failure loads typical of the structure, can lead to structural failure when applied cyclically.

**Figure 1:**
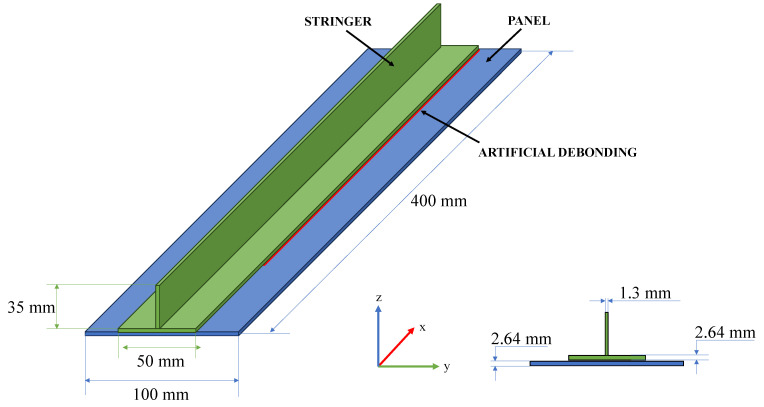
Scheme of the investigated panel.

The panel investigated in this study consists of a skin, 400 mm long in the longitudinal direction (x direction in Figure 1) and 100 mm wide in the transverse direction (y direction in Figure 1), with a quasi-isotropic stacking sequence of [45,90,0,−45] 2 s. Each individual ply has a thickness of 0.165 mm, resulting in a total thickness of 2.64 mm for the skin. The stringer, with a T shape cross section, is positioned at the centre of the skin and characterised by a foot stringer that is 50 mm wide and 2.64 mm thick, with a stacking sequence of [45,90,0,−45,−45,0,90,45,0,90,90,0,0,90,90,0]. The web is 35 mm in height (z direction in Figure 1), and 1.3 mm thick, with a cross-ply layup of [0,90,90,0] s. A schematic representation of the panel is reported in Figure 1, where the red component represents the artificial disbond introduced between the skin and stringer.

## 3. Experimental Methods

The manufacturing process of the single stiffened panel, composed of carbon fibre/epoxy resin and characterised by an artificial debonding at the skin–stringer interface, involves several critical steps to ensure precision and structural integrity. Firstly, ad hoc tooling has been designed using Computer-Aided Design (CAD) software, specifically SpaceClaim. Its configuration consists of three aluminium blocks, each equipped with four fasteners. The aluminium alloy Al 6082 T6 has been used for the production of the tooling, whose properties are listed in Table 1. The panel is placed inside the cavity and secured along the web by the four fasteners; then, the skin is placed on top of it and anchored using the top plate.

**Figure 2:**
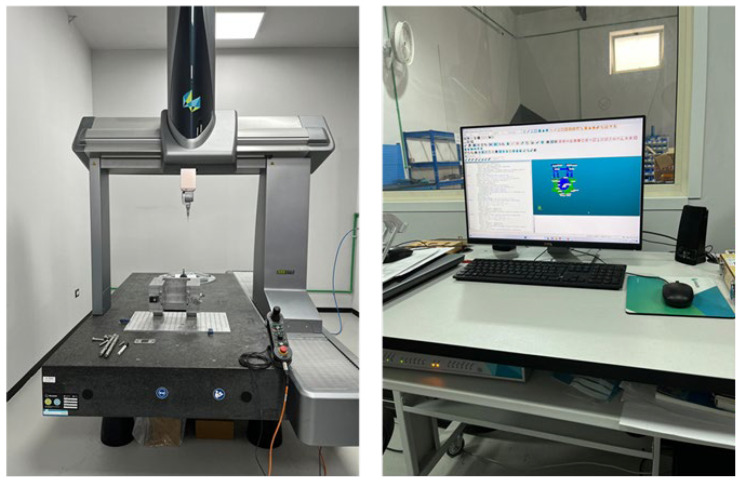
CNC machine and software.

All the activities have been carried out according to the procedures defined by the EN 9100 Quality Management System [26]. Figure 2 shows the employed CNC machine with the correlated software.

**Figure 3:**
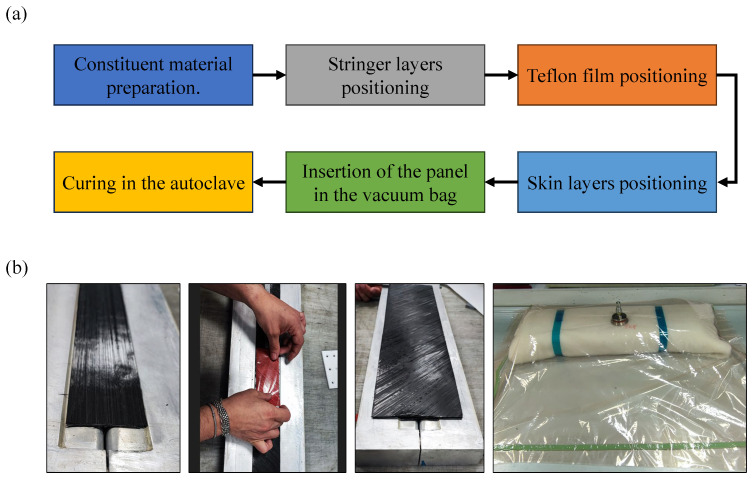
(**a**) Flowchart of the manufacturing of the panel, (**b**) images acquired during manufacturing process.

After the tooling production, the panel manufacturing process has been started. A Carbon Fibre/Epoxy resin material system has been used to manufacture the sample. The panel consists of a T-shaped stringer and a flat skin and are both co-cured in the autoclave to achieve optimal bonding and mechanical properties. The manufacturing process is described in the flowchart in Figure 3a, while the kay step pictures are shown in Figure 3b.

The panel has been cured in an autoclave. The curing step has been conducted under controlled pressure and at temperatures in the range of 120–180 °C.

Subsequently, the edges of the panels have been precisely machined by waterjet cutting. Water jet cutting has been selected as it is highly effective for composites, offering a combination of precision, quality, and versatility. Indeed, unlike thermal cutting methods, water jet cutting does not generate heat. This prevents thermal distortion, delamination, and degradation of the material properties.

**Figure 4:**
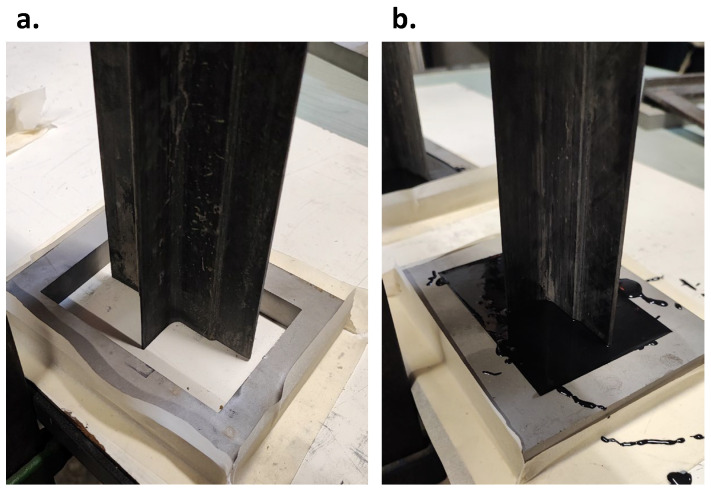
(**a**) Steel fixtures, (**b**) Potting.

Finally, potting has been performed. Potting helps to distribute the loads applied during experimental tests uniformly. Custom still fixtures have been produced to contain the potting material within the desired edge regions. These fixtures help in maintaining the shape and prevent the potting material from spreading. The image of the produced fixture is shown in Figure 4a.

The choice of the potting material has been based on the required mechanical properties, environmental resistance, and compatibility with the composite materials. SikaBiresin^®^ G519 (Epo 5019) epoxy casting resin with hardness 90 Shore D has been selected. The main characteristics of this resin are its high accuracy, good compressive strength, low shrinkage, good abrasion resistance, and high hardness. The resin is composed of two components:

- Component A: SikaBiresin^®^ G519, epoxy resin, filled, black.
- Component B: SikaBiresin^®^ G519, amine, unfilled, amber.

Component A is used with Component B SikaBiresin^®^ G519 for a long pot life. The image of the potting can be seen in Figure 4b. The potting material has been left to cure according to the manufacturer’s instructions. This process required over 16 h. After complete curing of the filler material, it was checked that the specimen was securely attached to the steel support, making sure that there was no displacement or separation during visual or tactile inspection.

The elastic properties of the considered Carbon Fibre/Epoxy resin material system and its interlaminar toughness have been measured by means of an extensive experimental campaign. Over 70 specimens have been fabricated for the characterisation of the material properties under both static and fatigue loading conditions. Tensile, compressive, and in-plane shear tests have been performed, according to the ASTM D3039 [27]—D3410 [28]—D3518 [29] standards, respectively, to determine the elastic moduli and the Poisson’s ratio. Double Cantilever Beam tests and End-notched Flexure tests have been carried out, according to ASTM D5528 [30]—D7905 [31], respectively, to measure the Mode I and Mode II interlaminar fracture toughness. The properties are listed in Table 2.

**Table 1:** Al 6082 T6 chemical composition and properties.

Spec: BS EN 573-3:2009 [31]	% Present
*Manganese* (*Mn*)	0.40–1.00
*Iron* (*Fe*)	0.0–0.50
*Magnesium* (*Mg*)	0.60–1.20
*Silicon* (*Si*)	0.70–1.30
*Copper* (*Cu*)	0.0–0.10
*Zinc* (*Zn*)	0.0–0.20
*Titanium* (*Ti*)	0.0–0.10
*Chromium* (*Cr*)	0.0–0.25
*Other* (*Each*)	0.0–0.05
*Others* (*Total*)	0.0–0.15
*Aluminium* (*Al*)	Balance
**Physical Property**	**Value**
*Density*	2.70 g/cm^3^
*Melting Point*	555 °C
*Thermal Expansion*	24 × 10^−6^/K
*Modulus of Elasticity*	70 GPa
*Thermal Conductivity*	180 W/m·K
*Electrical Resistivity*	0.038 × 10^−6^ Ω·m

**Table 2:** Material properties.

Spec: BS EN 573-3:2009	% Present
**Property**	**Value**
*E*_1_	122 GPa
*E*_2_	6.265 GPa
*G*_12_	4.649 GPa
*ν*_12_	0.3008
*ν*_23_	0.02
*G_Ic_*	258.82 J/mm^2^
*G_IIc_*	1928.4 J/mm^2^

The experimental investigation of the fatigue delamination propagation has been carried out by combining an experimental compressive test under load control with the use of lock-in thermography. The latter has been set up to capture thermal images of the panel at both maximum and minimum compressive load during specific fatigue cycles. It is worth specifying that preliminary numerical analyses have been performed to accurately select the load values and to estimate the fatigue cycle at which delamination advances.

**Figure 5:**
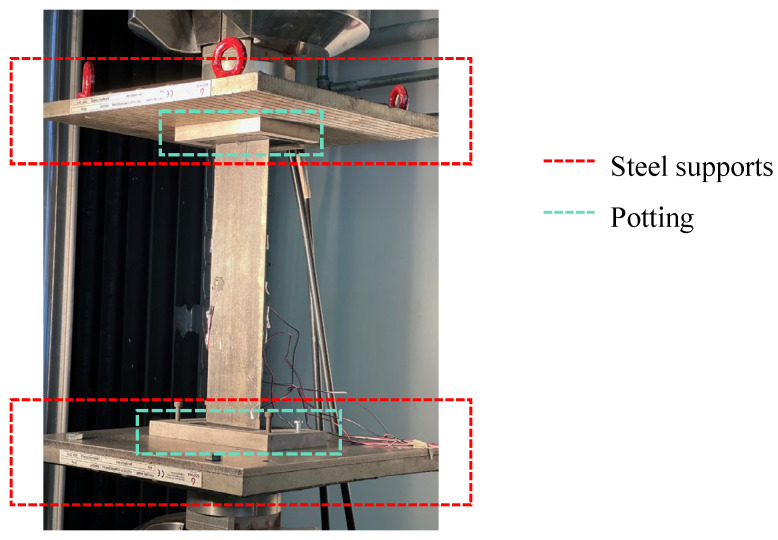
Positioning of the panel in the testing machine.

The compressive fatigue test has been performed with an MTS Landmark^®^ servo-hydraulic testing machine Model 370.50, equipped with a load cell with a maximum capacity of 500 kN. Test and monitoring software MPT/793 has been used to acquire and record the load and displacement data during the test execution. This data can be visualised in real-time in the form of graphs and subsequently analysed to determine the mechanical properties of the tested material. The machine is equipped with hydraulic grips needed to hold the sample firmly during the test. Two steel supports have been inserted into the machine grips (the upper and lower grips) to perform the compressive test, as can be seen in Figure 5. The steel supports provide solid and uniform surfaces on which to place the test specimen. During the compression test, it is essential that the applied force is uniformly distributed over the specimen to obtain accurate results. If the specimen is fixed directly in the grips without the supports, unwanted deformations or non-uniform force distribution may occur, affecting the test results. Moreover, supports act as a prevention of specimen damage. Indeed, using a steel holder can protect the structure from damage caused by grips, which can exert significant and rapid forces during installation and testing.

The panel has been placed on top of the steel supports so that the parts with potting are well fixed, as shown in Figure 5. Orientation must be precise to avoid any misalignment that could affect the test results. The panel has been fixed to the test machine supports by using appropriate fasteners to be sure that the panel cannot move or slip during the test.

The experiment was conducted at room temperature, with an average of 11.7 °C and a humidity level of 76.5%. A first quasi-static compression test has been carried out in displacement control. The strain gauges applied on the panel have been connected to the acquisition system for the strain recording. Subsequently, a compressive fatigue test was conducted under force control. The maximum and minimum force limits that the machine will apply during the test have been set to ensure the safety and integrity of the panel under the test. For this purpose, safety parameters have been established on the machine to control the maximum range of motion of the crosshead. This precaution is critical to avoid sudden and catastrophic failure of the panel during the test. Specifically, during a force-controlled test, the machine adjusts the position of the crosshead to maintain a constant load on the specimen, even if structural failures occur that change the stiffness of the specimen. This control mode helped avoid situations where the panel could be catastrophically crushed under high loads, increasing safety and ensuring that the test was conducted under controlled and safe conditions.

As already remarked, the panel has been subjected to a force-controlled compression-compression fatigue test. Two parameters have been set: a frequency of 2 Hz to prevent the overheating of the specimen, which could alter its elastic properties and a stress ratio (R) of 10. This implies that the minimum compressive load applied to the panel is always 10% of the maximum load applied.

**Figure 6:**
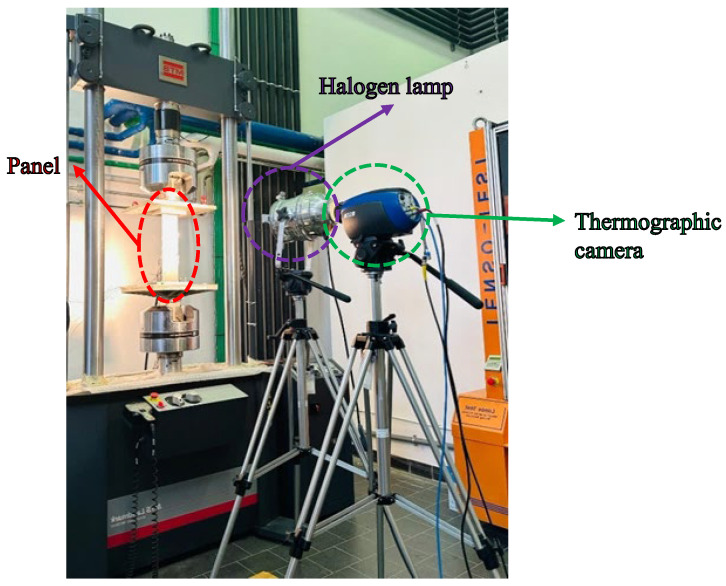
Thermographic system.

The debonding propagation during the test has been monitored using the Lock-in thermography system. Phase thermography maps are generated during the analysis of a modulated heated material. In this technique, the surface of the material is heated using a heat source that varies at a specific (modulated) frequency. The thermal response of the surface is then captured by an infrared camera. Active thermography, more specifically Lock-In Testing, has been used in this work to monitor the debonding propagation during fatigue cycles. The Lock-In system (in Figure 6) consists of a FLIR SC5500 high-performance thermal imaging camera that can detect and record temperature changes on the surface of the composite panel and one halogen lamp. The latter is controlled by a function generator, which modulates the lamp power by following a low-frequency (typically sinusoidal) signal. This means that the lamp emits light and heat in a time-varying manner, creating a modulated heat wave that propagates in the material under test. Modulated heat penetrates the material and travels through it. Due to the thermal properties of the material (such as thermal conductivity and diffusivity), the heat wave becomes attenuated and out of phase with respect to the original heat source as it propagates. The thermal imaging camera captures the thermal wave re-emitted from the surface of the material. This re-emitted thermal response contains information about the propagation of the thermal wave within the material. The system software uses an algorithm based on the Fast Fourier Transform (FFT) to analyse the captured thermal signal. The FFT decomposes the complex signal into a series of fundamental sinusoidal and harmonic components, allowing reconstruction of the modulated thermal wave that was re-emitted from the material. The next step is to compare the phase of the exciting thermal wave (the one generated by the halogen lamp) with the phase of the re-emitted thermal wave. The phase difference between these two waves provides crucial information. The amount of phase shift depends on the thermal properties of the material and the presence of any anomalies (such as defects, inclusions, or delaminations). Areas with damage or defects will have a different phase shift than undamaged areas.

The lock-in thermal imaging system has been positioned with respect to the test machine considering three factors: the thermal imaging camera field of view, the required spatial resolution, and operational safety. In general, the thermal imaging system has been positioned at a distance such that the camera has been able to fully frame the area of the panel under test, without compromising the resolution of the thermal images. The distance used for this application has been about 1.5 m from the panel. Furthermore, it has been considered that the halogen lamp used to heat the panel was placed to uniformly distribute the heat over the entire surface of the panel, without creating localised hot or cold regions. Finally, it has been verified that the thermal imaging system and lamp were securely positioned. The thermal imaging camera must be stably mounted, and the operator must maintain a safe distance from operating equipment, avoiding interference and risks.

**Figure 7:**
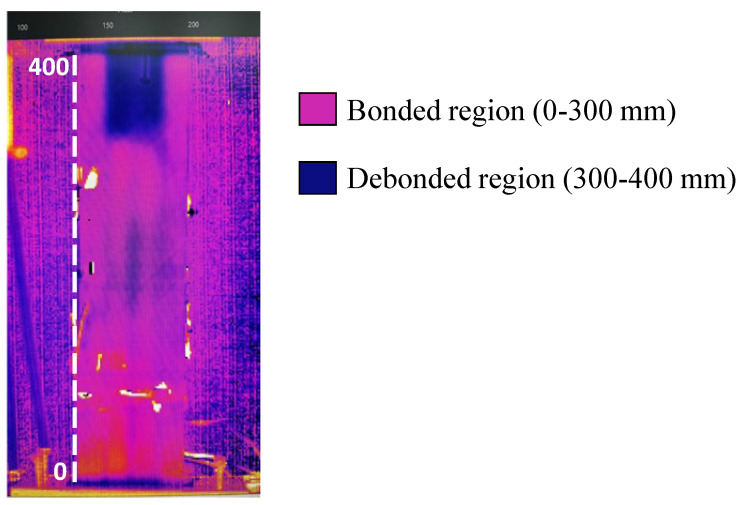
Phase map of the panel prior to fatigue test.

A series of preliminary tests are essential in order to properly determine the frequency of the modulated heating wave that will allow the area of the T-shaped spar with the best contrast to be identified. This area will be the reference map for the detection of defects from the thermographic images that will be acquired during the fatigue test. The reference phase map obtained at zero loading is shown in Figure 7. Phase maps have been acquired using the same excitation frequency of the halogen lamp, each time after a certain number of fatigue cycles, by interrupting the test.

Finally, the entire test rig, including the testing machine, thermal imaging camera, and lamp, has been surrounded by protective glass. This system is necessary to protect the operators from any debris that might be ejected during the test, especially in situations of structural breakage.

### 3.1. Pre-Crack Experimental Test

A pre-crack test has been performed on the structure. This is an experimental procedure used to check an initial crack in a structure before performing subsequent tests, such as fatigue or fracture tests. The main objective of the pre-crack test has been to ensure that the initial crack was well-defined and reproducible, allowing the behaviour of the structure to be accurately studied. This type of test is particularly useful for checking structural stiffness. In fact, it is used to ensure that the structure has the expected mechanical properties, as in the case of a delamination in a composite.

This test has been conducted under displacement control at 0.25 mm per minute. After the test, a thermographic inspection was performed to verify that there was no further advancement of the delamination.

**Figure 8:**
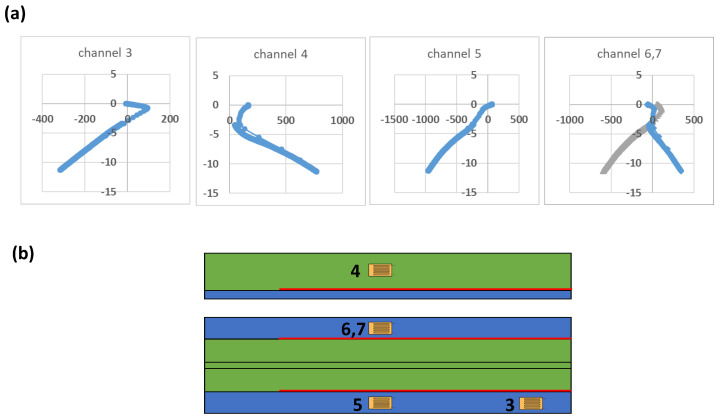
(**a**) Strain–Load Relationship in the Pre-Crack Test, (**b**) location of strain gauges.

The specimen has been equipped with strain gauges, in the positions shown in Figure 8b, to monitor the deformations under varying loads. Specifically, with the back-to-back strain gauges, it is possible to verify the buckling load, which is around 4.5 kN. The positioning of the strain gauges has been determined based on preliminary numerical analyses conducted prior to the tests, in order to identify the most significant locations.

### 3.2. Fatigue Test

For the fatigue test, a strategy of gradually increasing the applied load has been adopted. This method is commonly used in fatigue testing to evaluate the strength and durability of materials under cyclically increasing loads. This technique allows the gradual identification of material limits. Indeed, instantaneous damage, that could lead to catastrophic rupture of the panel, is avoided. Moreover, it allows the identification of the damage initiation load. Indeed, gradually increasing the load allows us to monitor the behaviour of the material and identify the point at which signs of degradation, such as the propagation of delamination, occur. This technique can also replicate realistic conditions. In fact, in real operating scenarios, loads on aircraft structures can vary over time. The considered loads are schematised in Table 3.

**Figure 9:**
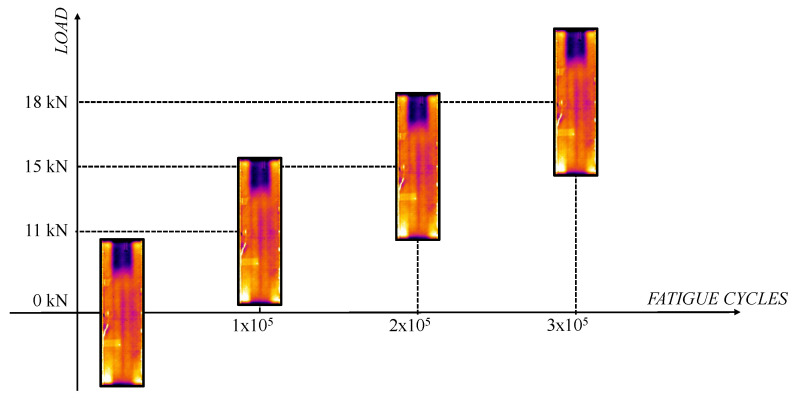
Evolution of debonding in the first 300,000 fatigue cycles considering variable applied load.

Among the phases, non-destructive inspections, using thermography, have been carried out to detect sub-surface damage that may not be visible. For the first 3 × 10^5^ cycles, performed at the loads shown in Table 3, thermographic images have been acquired at intervals of 1500 cycles. No evidence of damage has been found, as illustrated in Figure 9, which shows the thermographic images acquired at 1 × 10^5^, 2 × 10^5^, and 3 × 10^5^ fatigue cycles, performed at three different loads, as reported in Table 3, compared with the initial image at 0 cycles. The images appear identical as delamination does not show any propagation during these first three tests.

**Figure 10:**
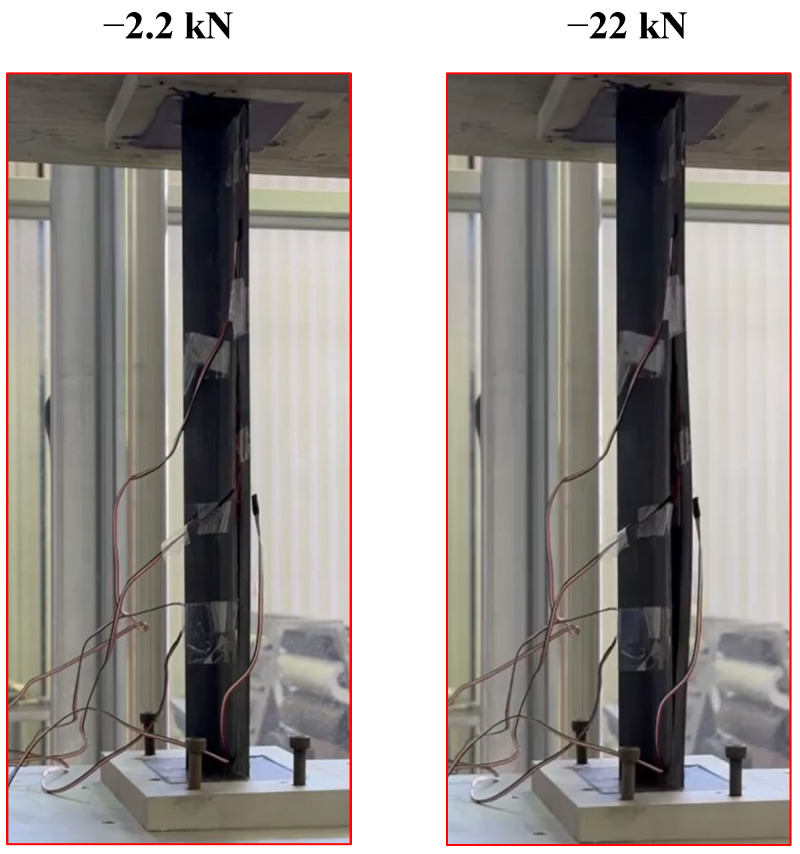
Panel under compression.

**Figure 11:**
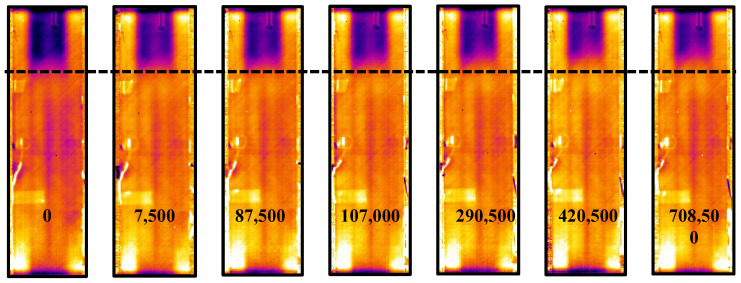
Thermographic images were acquired during the fatigue test.

Subsequently, the load has been increased to a maximum load of 22 kN and a minimum load of 2.2 kN. In Figure 10, the panel under compression can be observed, both at the minimum applied load P_min_ and the maximum applied load P_max_. Lock-In tests have been conducted every 1500 cycles until the onset of delamination, and subsequently every 5000 cycles. To facilitate the thermographic acquisition, the test was paused for 60 s while the specimen was subjected to the minimum load (P_min_). The thermographic images acquired showed that the debonding starts after 7500 fatigue cycles, as can be observed in Figure 11, which displays the debonding propagation at different fatigue cycles.

The thermographic images given in Figure 11 visually show damage propagation. However, filters should be applied to reduce the noise and improve the contrast between delaminated and undamaged areas. Indeed, quantification of the evolution of the delaminated area is difficult with the images in Figure 11.

## 4. Calculation of Delaminated Area

Post-processing of the phase maps has been necessary to assess the propagation of the debonding damage. Indeed, filtering thermographic images to obtain phase maps is an advanced technique for extracting detailed and specific information that is not visible in raw thermal images, thereby improving the analysis and understanding of thermal phenomena. Hence, the area involved in the evolution has been isolated by applying a filter able to separate the still bonded area from the rest of the specimen. This has been conducted by selecting an appropriate threshold value for all phase maps. The filter threshold value is an essential parameter in thermal image processing and must be chosen carefully according to the specific application and nature of the data. The correct choice of the threshold allows meaningful information to be extracted and thermal image analysis to be improved.

**Figure 12:**
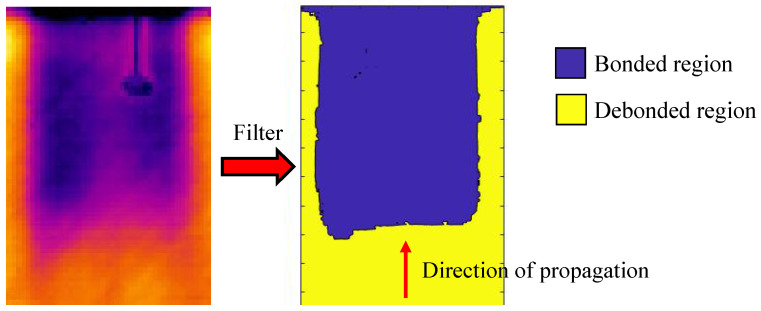
Filtered thermographic image of the panel prior to fatigue test.

Two different colours have been associated with each area: the bonded one and the rest of the sample. Figure 12 shows the images of the panel prior to the testing (cycle 0) with the applied filter. In the figure, the direction of debonding propagation is indicated.

Figure 12 shows the panel prior to the fatigue test, as already shown in Figure 7. However, it is clear that after the application of the filter, the distinction between the bonded and damaged regions is more pronounced and easier to detect.

**Figure 13:**
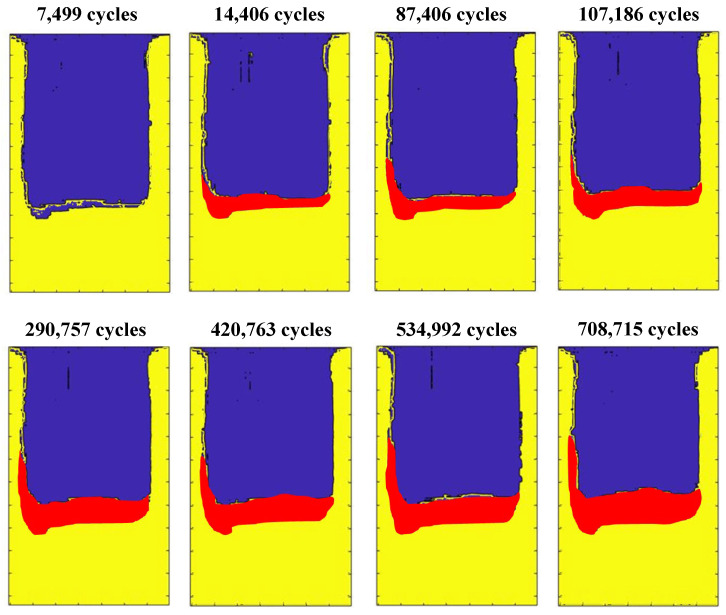
Debonding propagation at different fatigue cycles.

The images in Figure 13 have been obtained by superimposing the thermographic phase map obtained at a specific number of cycles with the thermographic phase map at cycle 0. Among the acquired phase maps, Figure 13 displays the cycles at which visible propagation of debonding has been observed. The red zone represents the debonding growth.

**Figure 14:**
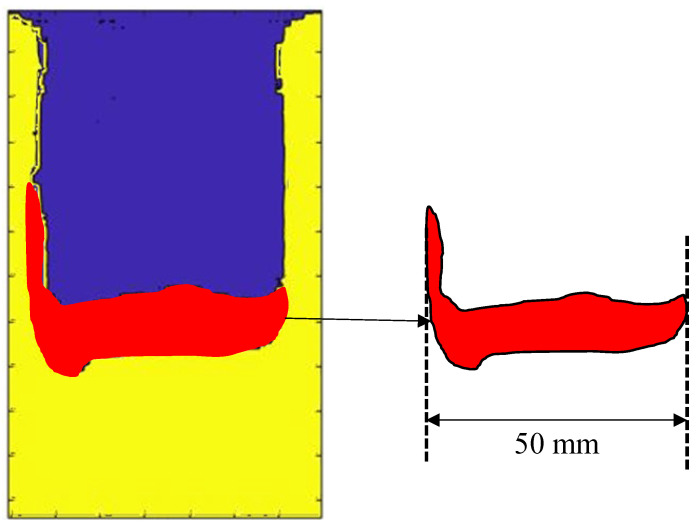
Debonded area calculation.

Phase maps in Figure 13 have been essential for calculating the increase in debonded area. Specifically, the number of pixels that constitute the object of interest in the image has been calculated. The key steps have been as follows:

- Isolate the image of which the area needs to be estimated, as shown in Figure 14.
- Use software to count the pixels that represent the object.
- Multiply the number of pixels by the area represented by a single pixel. If the resolution of the image is known (e.g., pixels per millimetres), the pixels can be converted to actual units of measurement (e.g., square millimetres).

ImageJ digital image processing software has been used to estimate the debonded area at different fatigue cycles. ImageJ is written in Java and is freely available and in the public domain, no license is required. The procedure for the area calculation has included the opening of the different area images in ImageJ software (version 1.54), and the definition of the scale using a known measure within the image, in this case, the width of the stringer has been used as shown in Figure 14, and finally the use of the command to count the pixels and calculate the area of the selected object.

**Figure 15:**
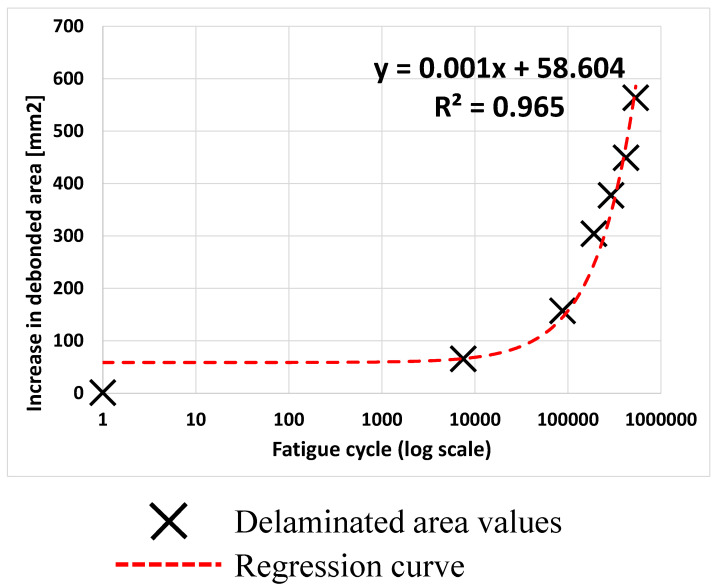
Increase in debonded area as a function of the fatigue cycles.

The result of this procedure for area calculation is shown in Figure 15, where the curve representing the increase in debonded area as a function of the fatigue cycles is reported. A log-scale has been used on the x-axis. The black x represents the values of the delaminated area calculated with the thermographic phase maps, while the dashed red line is a regression curve that highlights the trend of the debonding over the number of fatigue cycles.

According to the curve in Figure 15, debonding propagation begins gradually, indicating a stable initial phase. As the number of cycles increases, the propagation accelerates and becomes unstable, approximately around 1 × 10^5^ cycles. During the stable phase, incremental crack growth per cycle is relatively slow due to initial material strength and intact microstructure. As the number of cycles increases, microstructural degradation accumulates, leading to matrix cracking, fibre–matrix debonding, and secondary crack formation. The accumulation of damage causes acceleration of propagation, which becomes unstable and indicates the transition to a critical threshold. Beyond this threshold, material integrity deteriorates more rapidly due to the interactive effects of various damage mechanisms, leading to rapid growth of the debonding area. This behaviour emphasises the importance of detection and early intervention to avoid catastrophic failure.

## 5. Conclusions

In this study, an experimental approach combining compressive fatigue testing with lock-in thermography has been employed to investigate the propagation of fatigue-driven debonding in composite stiffened panels. The analysis of thermographic phase maps captured during fatigue cycles has allowed for the precise monitoring of debonding evolution between the skin and stringer. This non-destructive method has proven effective in quantifying both the extent and shape of the damage, providing valuable data for future validation of numerical models.

A single stringer stiffened panel has been manufactured, containing an artificial debonding, and tested under fatigue compression. The following key findings have emerged from the research:

1. A pre-crack test has been conducted, revealing panel buckling at 4.5 kN.
2. During the first 300,000 cycles conducted at three different load levels, no damage progression has been observed analysing the thermographic images.
3. With a maximum load of 22 kN, delamination propagation has been observed as early as 7500 cycles. The panel has been tested for up to 1 million fatigue cycles.
4. The use of thermographic phase maps has been crucial in identifying and quantifying the growth of delaminated areas. These maps provided a detailed view of the initiation and propagation of the damage.
5. The progression of debonding has been initially stable, with a gradual increase in the area involved in propagation.
6. After a certain threshold of fatigue cycles, at almost 1 × 10^5^ cycles, the propagation of debonding accelerated, indicating a transition to an unstable phase.

The results contribute to the development of more damage-resistant composite structures. Understanding debonding behaviour under fatigue conditions allows better prediction of service life and informs maintenance schedules, improving the safety and reliability of composite components in aerospace and other industries.

## Figures and Tables

**Table 3:** Increasing cyclic load applied to the structure.

P_min_	P_max_	Elapsed Cycles
1.1 kN	11 kN	100,000
1.4 kN	14 kN	100,000
1.8 kN	18 kN	100,000
2.2 kN	22 kN	1,000,000

## Data Availability

The raw/processed data required to reproduce these findings are available on request to the corresponding author.
